# GC-STCL: A Granger Causality-Based Spatial–Temporal Contrastive Learning Framework for EEG Emotion Recognition

**DOI:** 10.3390/e26070540

**Published:** 2024-06-24

**Authors:** Lei Wang, Siming Wang, Bo Jin, Xiaopeng Wei

**Affiliations:** 1 School of Software Technology, Dalian University of Technology, Dalian 116024, China; wanglei2611@mail.dlut.edu.cn; 2 School of Information and Communication Engineering, University of Electronic Science and Technology of China, Chengdu 611731, China; 202122010706@std.uestc.edu.cn; 3 School of Innovation and Entrepreneurship, Dalian University of Technology, Dalian 116024, China; 4 School of Computer Science and Technology, Dalian University of Technology, Dalian 116024, China

**Keywords:** EEG, emotion recognition, contrastive learning, noise reduction, Granger causal

## Abstract

EEG signals capture information through multi-channel electrodes and hold promising prospects for human emotion recognition. However, the presence of high levels of noise and the diverse nature of EEG signals pose significant challenges, leading to potential overfitting issues that further complicate the extraction of meaningful information. To address this issue, we propose a Granger causal-based spatial–temporal contrastive learning framework, which significantly enhances the ability to capture EEG signal information by modeling rich spatial–temporal relationships. Specifically, in the spatial dimension, we employ a sampling strategy to select positive sample pairs from individuals watching the same video. Subsequently, a Granger causality test is utilized to enhance graph data and construct potential causality for each channel. Finally, a residual graph convolutional neural network is employed to extract features from EEG signals and compute spatial contrast loss. In the temporal dimension, we first apply a frequency domain noise reduction module for data enhancement on each time series. Then, we introduce the Granger–Former model to capture time domain representation and calculate the time contrast loss. We conduct extensive experiments on two publicly available sentiment recognition datasets (DEAP and SEED), achieving 1.65% improvement of the DEAP dataset and 1.55% improvement of the SEED dataset compared to state-of-the-art unsupervised models. Our method outperforms benchmark methods in terms of prediction accuracy as well as interpretability.

## 1. Introduction

As a crucial component of affective computing, emotion recognition has garnered increasing attention from scholars in recent years and has emerged as a significant research topic at the intersection of neuroscience, psychology, computer science, and artificial intelligence [1]. Broadly speaking, emotion recognition methods can be categorized into two groups: one based on non-physiological signals such as speech, text, facial expressions, etc. [2,3], and the other based on physiological signals like electroencephalogram (EEG), electrocardiogram (ECG), EMG, etc. [4,5,6]. Physiological signals directly reflect the brain’s state under different emotions and are considered to be more objective. With advancements in EEG acquisition equipment and technology, EEG has become the preferred method for studying the brain’s response to emotional stimuli.

Firstly, the mining of complex spatial topological relationships among EEG channels poses a significant challenge. The EEG signals are captured through multiple electrode channels that collectively form the spatial structure of these signals. Existing studies often determine channel spatial topological relationships solely based on the physical distance between electrodes or channel correlation, as exemplified by GCNN, P-GCNN, and GCNs-Net [7,8,9]. However, previous research has demonstrated that different brain electrical channels frequently exhibit intricate causal relationships. Therefore, simple undirected graph modeling struggles to accurately depict the complex information transmission between these channels [10,11]. Consequently, we argue that directed causal graph modeling can effectively capture the causal relationship between channels.

Secondly, high levels of noise interference constitute the primary factor contributing to model overfitting in emotion recognition tasks. During the process of EEG data collection, it is often subject to interference from internal emotions and external devices, resulting in a low signal-to-noise ratio characteristic of EEG data. Furthermore, the high temporal resolution of EEG signals necessitates a time-consuming and labor-intensive sample labeling process. Most supervised or semi-supervised methods are ineffective when dealing with limited labeled training data.Previous studies, such as SGMC and CLISA [12,13], employed video alignment to obtain similar samples for constructing positive sample pairs. These studies utilized an unsupervised contrast learning framework to extract intrinsic features of specific emotional EEG signals, thereby enhancing the model’s generalization capability. However, these methods still lack robustness against noise interference. Therefore, an effective strategy for constructing positive samples pairs temporally can further augment the model’s generalization ability.

To address the aforementioned challenges, this paper proposes a Granger causality based spatial–temporal contrast learning framework called GC-STCL. In the spatial dimension, we initially consider the EEG channel as a node and utilize the physical distance between sensors as edges to construct the original EEG graph structure. Subsequently, we introduced a graph augmentation method at the structural level by leveraging Granger causality. In contrast to approaches based on channel distance or correlation, our proposed method constructs a directed graph through rigorous Granger causality testing. This not only captures the causal relationships between brain electrical channels but also eliminates unnecessary connections, thereby enhancing the accuracy of emotion recognition. Furthermore, we employ a sampling strategy to select positive sample pairs from individuals who watched the same video. Finally, spatial feature extraction of EEG signals is achieved through multi-layer graph convolution connected by residual edges, followed by calculation of spatial contrast loss based on these extracted features. In terms of time dimension, we employ a frequency domain noise reduction data augmentation strategy to construct positive sample pairs. Differing from traditional time series data augmentation methods such as translation, masking and amplification, our proposed approach exhibits stronger resistance against noise interference. Furthermore, inspired by Granger causality tests, we propose using Granger–Former to capture time representations. Lastly, time comparison loss is calculated based on temporal characteristics.

To summarize, the primary contributions of this paper are as follows:

- We propose a spatial–temporal contrast learning framework (Granger-STCL) based on the Granger causality test, which effectively enhances the emotion recognition capability of EEG signals.
- We validate the significance of directed causal graph and temporal causal modeling. Furthermore, we introduce spatial–temporal positive sample pair construction strategies for enhancing causal graph and reducing frequency domain noise respectively, successfully improving the generalization ability of contrast learning.
- Extensive experimental results on two publicly available datasets demonstrate that GC-STCL outperforms the benchmark method in terms of prediction accuracy and interpretability.

## 2. Related Works

In this section, we comprehensively examine EEG-based methods for emotion recognition, with a primary focus on deep learning and contrastive learning approaches.

### 2.1. EEG-Based Emotion Recognition

As a result of the success of deep neural networks, deep learning-based emotion recognition has received more attention. Various deep learning methods have been utilized for this purpose. Zhang et al. [14] proposed a model which consists of two RNN layers to extract both temporal and spatial features from EEG for emotion recognition. Li et al. [15] introduced a self-attention network that captures spatial domain and frequency domain features from EEG by utilizing a parallel convolutional neural network (PCNN) layer. Chen et al. [16] presented layered bidirectional gated cycle unit (GRU) networks to mitigate the impact of long-term non-stationarity in EEG.

In order to investigate the relationship between EEG channels and extract spatial features, the researchers employed graph convolutional networks (GCN) to capture the interrelationships among brain electrical channels by constructing EEG networks based on either physical proximity or functional connectivity between channels. Yin et al. [17] constructed the graph network using a Gaussian kernel function and extracted both spatial and temporal features through GCN combined with LSTM. Du et al. [18] integrated Cartesian distance between channels and prior knowledge to establish a graph network, from which they extracted EEG features utilizing GCN. Lin et al. [19] determined the relationships among EEG channels using a phase lag index (PLI), then extracted intra-channel and inter-channel features through 1D convolutional layers and Graph Attention Networks (GAT). Feng et al. [20] identified brain electrical channel connections via a Pearson correlation coefficient and achieved emotion recognition by employing GCN, Bi-LSTM, as well as an attention mechanism.

Despite the favorable outcomes achieved by these approaches, a majority of them construct EEG data using undirected graphs, thereby overlooking the directional information flow between EEG signals. However, it has been demonstrated in previous studies that there is a causal relationship among brain regions [21], which significantly contributes to extracting EEG features and enhancing accuracy in emotion recognition.

### 2.2. Contrastive Learning

Contrastive learning, a self-supervised algorithm that leverages unlabeled data to acquire representations, has found extensive applications in computer vision (CV) [22], natural language processing (NLP) [23], and bioinformatics [24], etc. In the domain of EEG emotion recognition, there have also been studies employing contrastive learning techniques. Mohsenvand et al. [25] proposed a contrastive learning framework, SeqCLR, based on SimCLR. The model augments the original data through various techniques such as masking, linear scaling, and Gaussian noise addition, etc. By employing both pre-training and fine-tuning stages, impressive emotion recognition results are achieved. Li et al. [26] proposed an innovative approach for emotion identification through EEG by employing a convolutional neural network and incorporating supervised contrastive learning (ECNN-C). The proposed model not only mitigates computational complexity, but also enhances the accuracy of emotion recognition. Guo et al. [27] proposed a contrastive learning approach that integrates multi-head attention mechanisms for unsupervised representation learning and feature fusion, aiming to enhance the accuracy of emotion recognition.

Currently, there is a paucity of research on EEG based emotion recognition utilizing the contrastive learning framework. Furthermore, the current research often overlooks the interdependence between EEG channels during feature extraction, and there is a dearth of studies on noise data processing. Consequently, there exists significant potential for further development of contrast learning in the domain of emotion recognition based on EEG signals.

## 3. Methodology

### 3.1. Problem Definition

**Figure 1:**
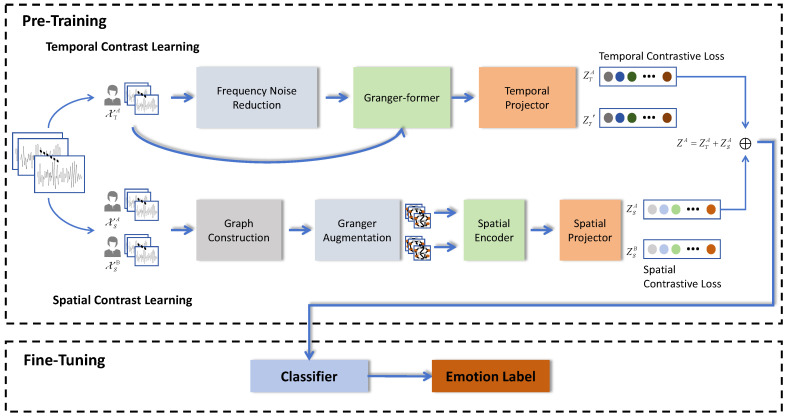
The overall structure of GC-STCL. The model comprises a pre-training process and a fine-tuning process. During the pre-training process, we employ the time contrast learning framework and the space contrast learning framework to extract temporal and spatial features from a small batch of input data, ultimately obtaining the feature representation of the input sample. In the fine-tuning process, classifiers are utilized to refine the pre-trained time and space encoders while conducting emotion classification training.

Given a time series $X\in R^{L\times V}$, where *L* represents the length of the input sequence and *V* represents the number of variables, our objective is to learn a nonlinear function $f_{\theta }$ that maps the input sequence X to the representation vector $Z\in R^{Fe}$, where Fe denotes the dimensionality of the representation. To achieve this goal, we propose two contrast learning frameworks for extracting EEG temporal and spatial features, respectively. In the temporal dimension, we employ a frequency noise reduction method to eliminate noise data from X, resulting in $X_{T}$. We consider both original time series data X and filtered data $X_{T}$ as positive sample pairs, encode them using Granger–Former, calculate contrast loss, and obtain the time domain representation of X, which is denoted as $Z_{T}$. For spatial dimension analysis, we collect EEG samples from two subjects watching identical video clips as positive samples. By constructing and enhancing graph data through the Granger causality test, encoding with ResGCN model, calculating contrast loss, we derive a spatial representation of X, which is referred to as $Z_{S}$. Finally, we combine these representations by summing them up as follows: $Z=Z_{T}+Z_{S}$. The overall structure of the model is presented in Figure 1.

### 3.2. Spatial Contrast Learning

#### 3.2.1. Graph Construction and Augmentation

In the spatial dimension, utilizing a graph structure to model EEG signals enables more effective utilization of information between EEG channels. Given a graph $G=\left(\Lambda ,E\right)$, Λ represents a set of nodes and *E* represents a set of edges. For EEG signals, $\Lambda_{c}$ denotes the channels of the signals. Let $A\in R^{C\times C}$ represent the adjacency matrix of *G*, which can depict the topology structure of EEG signals. The EEG graph can be defined as $G=\left(\Lambda ,E,A\right)$. According to previous studies [28], the correlation between EEG channels exhibits a negative relationship with the square of their Euclidean distance $d_{ij}$.

(1) $$dist\left(i,j\right)=\frac{\theta }{d_{ij}^{2}}$$

The adjacency matrix can be represented as follows:

(2) $$A_{ij}=\left\{\begin{array}{c} 1,\;dist\left(i,j\right)\geq \delta \\ 0,\;otherwise \end{array}\right.$$

where θ is the calibration constant and δ is the hyperparameter, which is used to remove the less correlated channels.

During the construction of the aforementioned adjacency matrix, the consideration of directionality in information transmission was omitted. Hence, this paper proposes a data enhancement method based on the Granger causality test to construct an asymmetric adjacency matrix that accurately reflects the directional relationships between EEG channels and improves emotion recognition accuracy [21,29]. For two EEG channels *i* and *j*, with their respective time series data denoted as $X_{i}=\left\{x_{i}^{1},x_{i}^{2},\cdots ,x_{i}^{L}\right\}$ and $X_{j}=\left\{x_{j}^{1},x_{j}^{2},\cdots ,x_{j}^{L}\right\}$, the fundamental concept behind the Granger causality test is that if combining the value of $X_{i}$ with that of $X_{j}$ yields better results than considering only the value of $X_{j}$, it indicates that $X_{i}$ has a Granger causal influence on $X_{j}$. The autoregressive model representing this relationship can be expressed as follows:

(3) $$\begin{array}{c} x_{i}^{t}=\sum_{p=1}^{P}\alpha_{1,p}x_{i}^{t-p}+\varepsilon_{1}^{t}\\ x_{j}^{t}=\sum_{p=1}^{P}\alpha_{1,p}x_{j}^{t-p}+\varepsilon_{2}^{t} \end{array}$$

The bivariate regressive model can be formulated as follows:

(4) $$\begin{array}{c} x_{i}^{t}=\sum_{p=1}^{P}\alpha_{11,p}x_{i}^{t-p}+\sum_{p=1}^{P}\alpha_{12,p}x_{j}^{t-p}+\varepsilon_{3}^{t}\\ x_{j}^{t}=\sum_{p=1}^{P}\alpha_{21,p}x_{i}^{t-p}+\sum_{p=1}^{P}\alpha_{22,p}x_{j}^{t-p}+\varepsilon_{4}^{t} \end{array}$$

The lag order of the model is denoted as *P*, the regression coefficients $\alpha_{1,p}$ and $\alpha_{2,p}$ represent the parameters for the univariate model, while $\alpha_{11,p},\alpha_{12,p},\alpha_{21,p},\alpha_{22,p}$ are the binary regression coefficients for the bivariate model. The prediction errors of the univariate autoregressive model, denoted as $\varepsilon_{1}^{t}$ and $\varepsilon_{2}^{t}$, have variances $\mathrm{var}\left(\varepsilon_{1}\right)$ and $\mathrm{var}\left(\varepsilon_{2}\right)$ respectively. Similarly, the prediction errors of the binary regression model, denoted as $\varepsilon_{3}^{t}$ and $\varepsilon_{4}^{t}$, have variances $\mathrm{var}\left(\varepsilon_{3}\right)$ and $\mathrm{var}\left(\varepsilon_{4}\right)$ respectively. In this context, Granger causality between the two channels can be defined.

(5) $$GC_{\Lambda_{j}\to \Lambda_{i}}=\ln\left(\frac{\mathrm{var}\left(\varepsilon_{1}\right)}{\mathrm{var}\left(\varepsilon_{3}\right)}\right)$$

(6) $$GC_{\Lambda_{i}\to \Lambda_{j}}=\ln\left(\frac{\mathrm{var}\left(\varepsilon_{2}\right)}{\mathrm{var}\left(\varepsilon_{4}\right)}\right)$$

After conducting the Granger causality test to enhance the aforementioned adjacency matrix, we obtain

(7) $$A_{ij}^{GC}=\left\{\begin{array}{c} 1,\;dist\left(i,j\right)\leq \tau \;and\;GC_{\Lambda_{j}\to \Lambda_{i}}>0\\ 0,\;otherwise \end{array}\right.$$

#### 3.2.2. Spatial Encoder

In order to extract EEG features for contrast learning, an encoder based on ResGCN and ResNet18 is designed in this paper [30,31]. It consists of nine residual blocks, each containing two GCN layers, all of which are connected by a residual. Given an input graph *G* with its representation at layer *l* denoted as $G_{l}$, the encoder first generates the residual graph representation $G_{l+1}^{res}$ at the next layer using a residual map *F* and then adds vertices to obtain the output $G_{l+1}$. Let *H* represent the underlying map of the encoder and $W_{l}$ denote the parameters to be learned at layer *l*. We obtain the following:

(8) $$G_{l+1}=H\left(G_{l},W_{l}\right)=F\left(G_{l},W_{l}\right)=G_{l+1}^{res}+G_{l}$$

The features extracted from each GCN layer are subsequently combined through a 1×1 convolutional layer and a maximum pooling layer, resulting in the acquisition of a comprehensive representation that integrates both global and local features.

(9) $$\tilde{G}=h\left(G\right)$$

**Figure 2:**
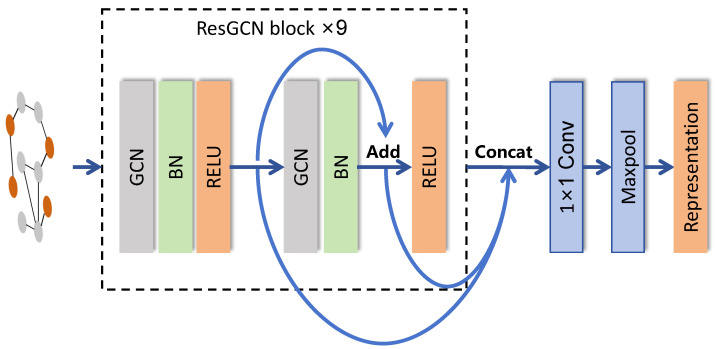
The architectures of spatial encoder.

The structure of the spatial encoder is illustrated in Figure 2.

#### 3.2.3. Projector

According to GraphCL [32], a nonlinear projection head is introduced between the encoder and the final contrast loss in order to project the encoded EEG signal learned by the encoder into a latent space for computing the contrast loss. In this study, we employ a Feed-forward Network (FFN) as the projection head. It comprises three 1×1 convolutional layers with Batch Normalization applied to each layer. Ultimately, this yields the feature representation.

(10) $$Z_{S}=FFN_{S}\left(\tilde{G}\right)$$

#### 3.2.4. Spatial Contrastive Loss Function

The sampling strategy is employed to select samples $X_{i}^{\prime }$ that have watched the same video clips as positive samples, corresponding to the representations $Z_{S,i}$ and $Z_{S,i}^{\prime }$, respectively, for sample $X_{i}$. The negative sample set $D_{i}^{S}$ is randomly drawn from samples that have watched other video clips. We employ a modified version of contrast learning inspired by MoCo to establish the spatial contrastive loss [33].

(11) $$L_{S}=\frac{1}{N}\sum_{i=1}^{N}-\log\frac{\exp(sim\left(Z_{S,i},{Z^{\prime }}_{S,i}\right)/\tau )}{\exp\left(sim\left(Z_{S,i},{Z^{\prime }}_{S,i}\right)/\tau \right)+\sum_{{Z^{\prime }}_{S,j}\in D_{i}^{S}}\exp(sim\left(Z_{S,i},{Z^{\prime }}_{S,j}\right)/\tau )}$$

(12) $$sim\left(Z_{S,i},{Z^{\prime }}_{S,i}\right)=\frac{Z_{S,i}\cdot {Z^{\prime }}_{S,i}}{∥Z_{S,i}∥∥{Z^{\prime }}_{S,i}∥}$$

where *N* denotes the number of samples and τ is the temperature parameter.

### 3.3. Temporal Contrast Learning

#### 3.3.1. Frequency Noise Reduction

The traditional methods for enhancing time series data typically employ time shift, masking, and amplitude scale, etc. [25]. However, these aforementioned techniques are not suitable for EEG signal prediction tasks. Firstly, EEG signals exhibit high complexity and individual specificity. Traditional data enhancement methods may disrupt the subtle patterns and correlations present in EEG signals, thereby significantly impacting the accuracy of prediction tasks. Secondly, EEG signals possess strong temporal dependencies. Enhancement techniques such as time shift or amplitude scale can alter the temporal relationships within the signal, distorting its inherent time dependence. Lastly, each component of an EEG signal potentially carries crucial physiological information regarding brain states. Methods like adding masks or introducing random noise may obscure or modify this critical physiological information, resulting in predictive models that fail to accurately interpret or identify specific brain states. Consequently, we propose a frequency domain noise reduction data enhancement strategy that is better suited for processing EEG signals.

Specifically, for a given input signal $X_{T}$, it is initially transformed into the frequency domain using the fast Fourier transform (FFT). Subsequently, noise reduction is accomplished by filtering out frequencies corresponding to the Top-K amplitudes, thereby obtaining the respective positive sample $X_{T}^{\prime }$.

(13) $$X_{T}^{\prime }=F^{-1}\left(SelectF\left(TopK\left(A\left(F\left(X_{T}\right)\right)\right)\right)\right)$$

where F denotes the FFT and $F^{-1}$ is its inverse, $F\left(X_{T}\right)\in R^{E}$, where E=⌊L/2⌋+1, SelectF() denotes the frequency that corresponds to the selected amplitude, $A\left(F\right)=\sqrt{F_{r}^{2}+F_{i}^{2}}$, $F_{r}$ and $F_{i}$ represent the corresponding real and imaginary parts, respectively.

#### 3.3.2. Granger–Former

To enhance the capture of temporal relationships in time series data, we propose extracting temporal EEG representations based on Granger causality and employ the Granger causality test instead of traditional self-attention mechanisms to depict causal connections among the data.

Transformer exhibits significant potential in time series prediction tasks [34]. However, conventional self-attention mechanisms can only capture the relationships between time points in time series modeling and fail to account for more intricate causal associations preceding or succeeding specific segments of time. Hence, we adopt the foundational framework of Transformer and substitute self-attention mechanism with Granger-attention to achieve temporal causality modeling. Specifically, for a given time series $X\in R^{L\times V}$, we first capture the time period after each time point with a length of *P* through a sliding window. If the length after the time point is less than *P*, it is padded with zeros. This results in obtaining $X_{P}\in R^{L\times P\times V}$. Subsequently, the queries $Q\in R^{L\times P\times Fe}$, keys $K\in R^{L\times P\times Fe}$, and values $V\in R^{L\times P\times Fe}$ are mapped through three linear layers. Finally, the attention relevance score is ultimately computed via a Granger causality test, thereby facilitating the comprehensive aggregation of values.

(14) $$\tilde{Z_{T}}=softmax\left(ReLU\left(Granger\left(Q,K\right)\right)\right)\cdot V$$

**Figure 3:**
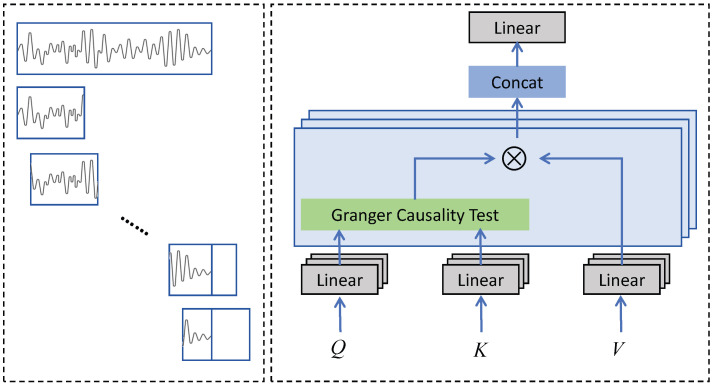
The architectures of Granger attention.

The calculation details of Granger() are presented in Section 3.2.1. Associations below zero are rectified to zero using the ReLU() activation function.The structure of Granger–Former is presented in Figure 3.

We also use the MLP as the temporal projector, facilitating the acquisition of a refined representation of the input signal in the time domain.

(15) $$Z_{T}=MLP_{T}\left(\tilde{Z_{T}}\right)$$

#### 3.3.3. Temporal Contrastive Loss Function

Similar to the calculation of contrast loss in the spatial dimension, we select sample ${X^{\prime }}_{T,i}$ after noise reduction as a positive pair for sample $X_{T,i}$. These samples are then subjected to Granger causality analysis to allow us to determine their corresponding weights, resulting in $Z_{T,i}$ and ${Z^{\prime }}_{T,i}$. From all samples, we randomly draw the negative sample set $D_{i}^{T}$. Finally, the temporal contrastive loss can be expressed as follows:

(16) $$L_{T}=\frac{1}{N}\sum_{i=1}^{N}-\log\frac{\exp(sim\left(Z_{T,i},{Z^{\prime }}_{T,i}\right)/\tau )}{\exp\left(sim\left(Z_{T,i},{Z^{\prime }}_{T,i}\right)/\tau \right)+\sum_{{Z^{\prime }}_{T,j}\in D_{i}^{T}}\exp(sim\left(Z_{T,i},{Z^{\prime }}_{T,j}\right)/\tau )}$$

The total loss of GC-STCL is formulated as follows:

(17) $$L=L_{S}+L_{T}$$

## 4. Experiments

To validate the model, we conducted extensive experiments on two widely used emotion recognition datasets, namely DEAP and SEED, and compared them with state-of-the-art methods. Additionally, we designed ablation experiments to verify the rationality of each component of the model and further discussed the role of frequency noise reduction and the Granger causality test in enhancing its performance.

### 4.1. Settings

#### 4.1.1. Datasets

DEAP [35]: The DEAP dataset comprises 32 channels of EEG signals and 8 channels of other physiological signals, which were recorded from 32 subjects during their observation of a series of 40 one-minute music videos. Each segment of the EEG data consists of a baseline period lasting for 3 s followed by a test signal lasting for 60 s. All the data were downsampled to a frequency of 128 Hz and processed using a bandpass filter with cutoff frequencies ranging from 4 Hz to 45 Hz. Subsequently, participants provided ratings on a scale from 1 to 9 regarding their emotional state after watching the videos, encompassing dimensions such as arousal, valence, liking, and dominance. In this paper, we employed a 1 s sliding window to partition the EEG signal into distinct segments totaling up to 63 segments. Subsequently, the average of the preceding 3 s baseline signal was subtracted from each 1 s emotion-related signal, serving as the input data. For each subject, we have access to a dataset comprising 2400 signals (40 videos, with each video containing 60 1 s signals), resulting in a total of 76,800 input samples. Consistent with previous research in this field, our specific focus lies in investigating arousal and valence for emotion recognition purposes. More specifically, scores equal to or above 5 indicate high arousal or high valence levels, while scores below 5 indicate low arousal or low valence levels.

SEED [36]: The SEED dataset consists of EEG signals recorded from 15 subjects while they observed 15 videos, each with an approximate duration of 4 min. Prior to the experiments, the videos were pre-classified as negative, neutral, or positive. The participants underwent three separate sessions with a one-week interval between them. Within a timeframe of 45 s after watching each video, the subjects rated their emotional responses. The collected EEG signals comprise a total of 62 channels and were downsampled to a frequency of 200 Hz before undergoing processing using a bandpass filter ranging from 0 Hz to 75 Hz. We implemented a data processing methodology similar to that employed in the DEAP dataset, resulting in 3394 1 s signals for each subject and yielding a cumulative total of 152,730 input samples (obtained from 15 subjects during 3 sessions).

#### 4.1.2. Baselines

For the DEAP dataset, we compared our model with state-of-the-art emotion recognition models in valence, arousal dimensions and valence and arousal classification. These models include LResNet [37], an emotion recognition method based on a deep residual network; MMResLSTM [38], a multimodal residual LSTM network for emotion recognition; ERDL [17], a GCN and LSTM network for emotion recognition; ACRNN [39], an attention-based convolutional RNN model; GLFANet [40], a global and local feature aggregation net. We also compared it with two self-supervised emotion recognition frameworks: GANSER, which is based on Generative Adversarial Network [41], and SGMC, which is based on based on group meiosis contrastive learning [12] for EEG-based emotion recognition.

Similar to the DEAP dataset, we selected the five most advanced supervised models and two self-supervised models for comparison on the SEED dataset. These include BiHDM [42], a bi-hemispheric discrepancy model; ResNet18-1D [31], a residual network consisting of 17 convolutional layers and 1 fully connected layer; DGCNN [43], a GCN and LSTM network; RGNN [44], a regularized graph neural network, and CLISA [13], a contrastive Learning method for learning Inter-Subject Alignment. Additionally, GLFANet [40] and SGMC [12] models, which serve as baseline models in the DEAP dataset, are also incorporated.

#### 4.1.3. Implementation Details

The experiment was conducted on the Geforce RTX 3090Ti GPU using the pytorch [45] framework. For optimization, we selected the Adam optimizer [46] with a learning rate of 0.001.The experimental setup we adopted bears a resemblance to that of SGMC [12]. During pre-training, we performed 3000 training sessions on two datasets with a batch size of 32. In the spatial contrastive learning framework, based on prior research experience [40], the calibration constant θ was set to 6 and was used to normalize the reciprocal distance between EEG channels to a range of (0,1). The hyperparameter δ was set to 0.1 in order to exclude channels exhibiting lower correlation. In the temporal contrastive learning framework, based on empirical evidence, we set the value of top-K to 20, indicating that we selected the frequencies corresponding to the top 20 largest amplitudes as representative samples after applying noise reduction. The hyperparameter τ for calculating contrast loss was set to 0.1. For fine-tuning, we used a batch size of 256 and trained for a total of 100 epochs. We employed a five-fold cross-validation methodology and calculated the average performance across all five validation iterations as our final experimental result.

### 4.2. Performance Comparison

**Figure 4:**
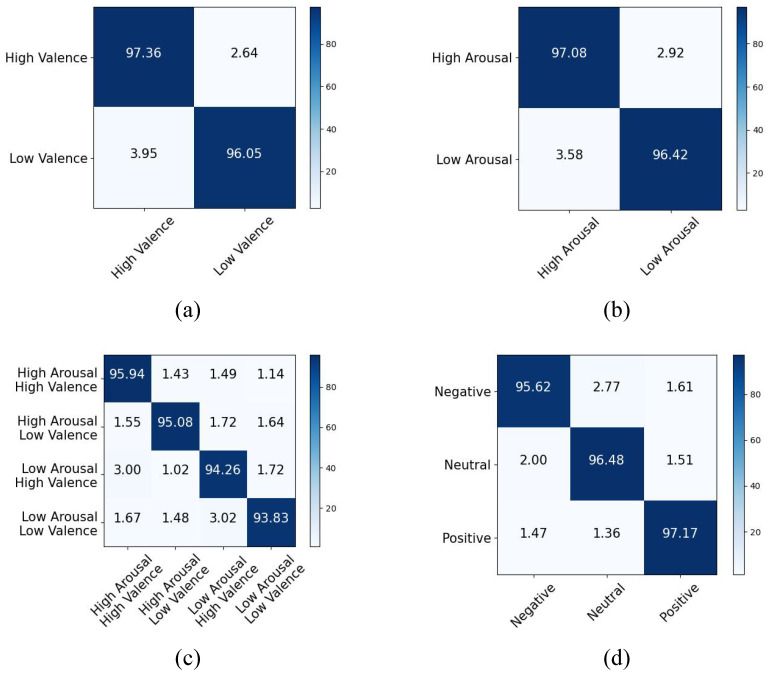
The confusion matrix of classification on DEAP and SEED. (**a**) Valence on DEAP; (**b**) arousal on DEAP; (**c**) valence and arousal classifications on DEAP; (**d**) SEED.

We employed classification accuracy to evaluate the model’s performance, and the corresponding results for both datasets are presented in Table 1 and Table 2. Additionally, Figure 4 depicts the confusion matrices of the model. The experimental results demonstrated that the proposed model achieved recognition accuracies of 96.79% and 96.81% for the valence and arousal dimensions, respectively, on the DEAP dataset, surpassing the performance of previous state-of-the-art models by 1.55% and 1.06%. The accuracy of the valence and arousal classifications reached 94.96%, demonstrating an increase of 1.65%. Moreover, for the SEED dataset, our model achieved an accuracy improvement of 1.55%, reaching an accuracy rate of 96.43%. The primary reason is that the model effectively captures both the temporal and spatial features of EEG. When extracting temporal features, signal noise is efficiently eliminated through frequency domain noise reduction, and Granger causality between time segments is extracted for each signal, which has not been considered in previous studies. The experimental results also validate the efficacy of this approach. In terms of extracting spatial features, the Granger causality test is employed to extract directed causality between brain electrical channels, thereby reflecting the information flow within the EEG signal in line with brain mechanisms. Simultaneously, leveraging a contrast learning framework enables better extraction of inherent characteristics specific to various emotions within the EEG signal and ultimately achieves the optimal emotion recognition performance.

Based on the confusion matrix results, our model has demonstrated excellent performance in accurately recognizing various emotions across both datasets, indicating the effectiveness of the contrast learning framework in extracting EEG features associated with diverse emotional states.

### 4.3. Ablation Study

In order to investigate the rationality of the model, a series of ablation experiments were conducted. Firstly, we individually eliminated the space feature extraction module and the time feature extraction module to validate their respective functions in this paper’s model. Additionally, to further examine the impact of each component in the model, we also removed the frequency noise reduction, Granger–Former, and Granger graph augmentation modules. When the frequency noise reduction module was omitted, we employed the data mask method to enhance the temporal dimension data; when excluding Granger–Former, we utilized a conventional transformer as the encoder; and when disregarding Granger graph augmentation, we applied a random edge dropping method to augment spatial dimension data, as depicted in Table 3. The experiment was conducted on two datasets, and the corresponding results are presented in Table 4.

The removal of the temporal feature extraction module led to decreases in the performance on the DEAP and SEED datasets of approximately 6.46% and 8.24%, respectively, while the removal of the spatial feature extraction module resulted in decreases of about 7.83% and 8.24%. These findings provide evidence of the crucial role played by simultaneous extraction of both temporal and spatial features in enhancing the emotion recognition efficacy of EEG signals.

When employing data masking instead of a frequency noise reduction module, the accuracy experiences reductions of approximately 4.51% and 4.44%, substantiating the efficacy of the proposed method in eliminating noise from EEG signals and enhancing emotion recognition accuracy, thus establishing it as an effective approach for data augmentation. The utilization of a transformer instead of Granger–Former results in a reduction in accuracy by approximately 4.64% and 4.42%, thereby substantiating the efficacy of Granger–Former in effectively extracting pertinent features between time segments of EEG signals, a facet that has been overlooked in prior studies on emotion recognition. When random edge deletion was employed instead of Granger causal graph augmentation, the accuracy exhibited decreases of approximately 3.82% and 3.44%. This decline may be attributed to the effectiveness of the Granger causality test in capturing the directional flow of information in EEG signals during spatial information extraction, aligning with brain mechanisms and facilitating more accurate extraction of spatial features between brain electrical channels, thereby enhancing recognition accuracy.

## 5. Discussion

### 5.1. Granger Causality Analysis

**Figure 5:**
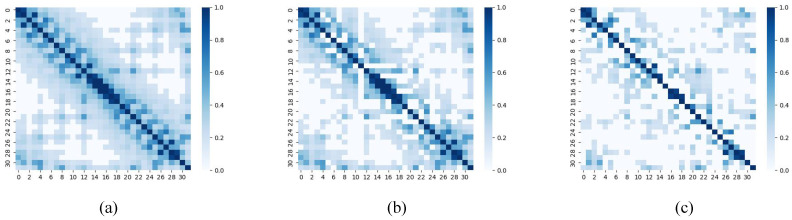
Heat map of the adjacency matrix before and after Granger causality augmentation. (**a**) Adjacency matrix without Granger causal augmentation; (**b**) augmented adjacency matrices incorporating Granger causality for emotions characterized by high arousal and high valence; (**c**) augmented adjacency matrices incorporating Granger causality for emotions characterized by low arousal and low valence.

The Granger causality test is employed during the process of spatial feature extraction to augment the initial symmetric adjacency matrix into an asymmetric matrix, thereby capturing the directional information flow within EEG signals. Taking the DEAP dataset as an example, a subject was randomly selected, and the EEG signals of two emotions (high arousal and high valence, low arousal, and low valence) were recorded after enhancement. The adjacency matrix heat map is presented in Figure 5. The adjacency matrix of high-arousal and high-valence emotions exhibits a greater number of connections compared to that of low-arousal and low-valence emotions, implying a higher level of complexity in information transmission within the former.

**Figure 6:**
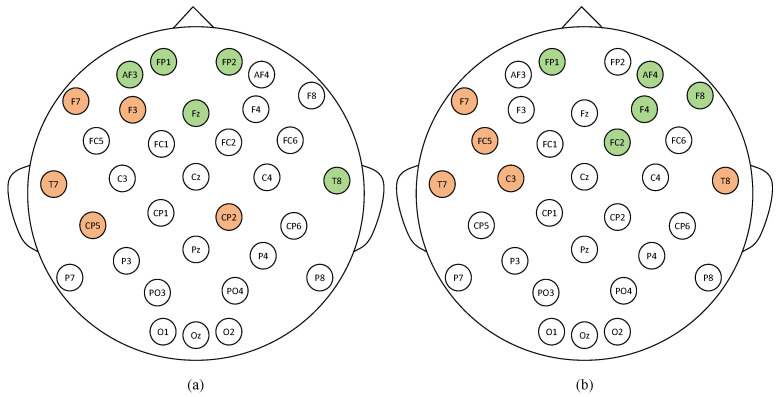
The channels exhibiting higher levels of activity in both emotional responses. (**a**) High arousal and high valence; (**b**) low arousal and low valence.

We also compute the disparity between the out and in degrees of each channel in these two emotions. When the positive difference of the channel is observed, it indicates that the channel serves as an input for information transmission, and vice versa. The top five channels for both information input and output are illustrated in Figure 6.

The color orange represents the outbound channel of information flow, while green signifies the inbound channel for information transmission. As depicted, the frontal and temporal lobes are closely related to emotions, and the flow of information is directional in different emotions, which aligns with previous research findings [47,48,49].

### 5.2. Noise Disturbance Experiment

**Figure 7:**
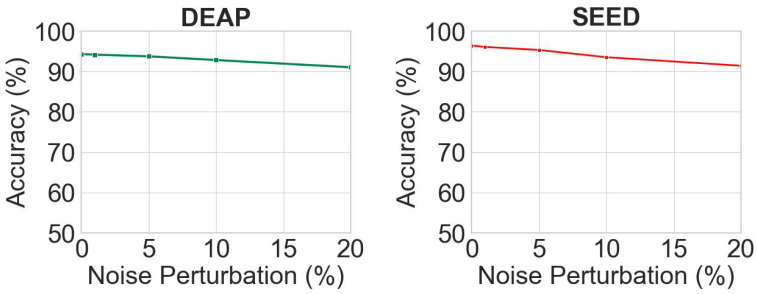
The results of the noise disturbance experiment.

In order to further validate the efficacy of the proposed frequency domain noise reduction module, we conducted a noise disturbance experiment on the DEAP dataset. Specifically, we applied noise perturbations of 1%, 5%, 10%, and 20% to the input signal during the time contrast learning stage, respectively. Subsequently, experiments were conducted on the DEAP and SEED datasets, and the corresponding results are illustrated in Figure 7.

The results demonstrate that the inclusion of a 20% noise disturbance in the data results in a reduction of merely 3.47% and 4.13% on the two datasets, thereby indicating the efficacy of frequency domain noise reduction approach in effectively eliminating EEG signal interference and consequently enhancing emotion recognition accuracy.

## 6. Conclusions

In this paper, we proposed a Granger causal-based data augmentation graph contrastive learning framework for emotion recognition. This framework constructs the graph structure as the input to use the relationship between the channels of the EEG signals. To reduce the influence of noise on emotion recognition while maintaining the graph structure, we chose the Granger causality test to enhance the input data, and then we trained a residual graph convolutional network to extract the features of the EEG signals.

To verify the validity of the proposed method, we conducted experimental tests on the DEAP and SEED datasets. The accuracies of the DEAP dataset for valence and arousal are 96.79% and 96.81% and the accuracy of SEED dataset is 96.43%. The experimental results show that the framework achieved state-of-the-art results for both datasets. We also performed ablation experiments to verify the validity of the various components of the framework.

Despite the commendable outcomes achieved using both datasets, the proposed method still exhibits certain noteworthy limitations. Firstly, it is important to acknowledge that the current public datasets are relatively small in size, encompassing a maximum of 32 subjects (DEAP). Consequently, this constraint may impede further advancements in the performance of this study. Furthermore, within the proposed contrastive learning framework in this paper, Granger–Former exhibits relatively high computational complexity, while spatial contrastive learning requires a large number of model iterations to ensure comprehensive sample training, resulting in increased computational costs. Finally, given the substantial inter-individual differences observed in EEG signals, it is imperative to validate the model’s performance across different subjects. These challenges collectively pose obstacles to practical implementation of the model.

In future research, it is imperative to construct a comprehensive dataset encompassing a larger number and diverse range of subjects in order to analyze the EEG signal characteristics across different emotional states among various populations. Furthermore, optimizing the model to reduce computational complexity, enhancing its generalization ability, and conducting subject-independent verification are crucial directions for future investigations.

## Figures and Tables

**Table 1:** Performances on DEAP.

Method	Accuracy (%)		
	Valence	Arousal	Valence & Arousal
LResNet (2018)	90.39	89.06	-
MMResLstm (2019)	92.87	92.30	-
ERDL (2021)	90.45	90.60	-
ACRNN (2020)	93.72	93.38	-
GLFANet (2023)	94.53	94.91	92.92
GANSER (2022)	93.52	94.21	89.74
SGMC (2023)	95.31	95.79	93.42
Proposed	**96.79**	**96.81**	**94.96**

**Table 2:** Performances on SEED.

Method	Accuracy (%)
BiHDM (2020)	93.12
ResNet18-1D (2021)	93.42
DGCNN (2018)	90.40
GLFANet (2023)	93.19
RGNN (2020)	94.24
CLISA (2022)	86.4
SGMC (2023)	94.96
Proposed	**96.43**

**Table 3:** The design of the ablation study.

Method	Temporal	Spatial	Noise Reduction	Granger–Former	Graph Augmentation
GC-STCL-temporal	×	√	×	×	√
GC-STCL-spatial	√	×	√	√	×
GC-STCL-noise	√	√	×	√	√
GC-STCL-gf	√	√	√	×	√
GC-STCL-ga	√	√	√	√	×

**Table 4:** The results of the ablation study.

Method	Valence and Arousal Classifications Accuracy (%) on DEAP	Accuracy (%) on SEED
GC-STCL-temporal	88.83/4.18	89.76/4.57
GC-STCL-spatial	87.52/3.45	88.48/4.10
GC-STCL-noise	90.68/3.38	92.15/4.37
GC-STCL-gf	90.55/3.29	92.16/3.39
GC-STCL-ga	91.33/3.23	93.11/3.30
**GC-STCL**	**94.96/2.70**	**96.43/2.99**

## Data Availability

Two publicly available datasets were analyzed in this study. These data can be found at the following address: http://www.eecs.qmul.ac.uk/mmv/datasets/deap/index.html (accessed on 20 July 2022), and https://bcmi.sjtu.edu.cn/home/seed/ (accessed on 16 March 2021).
